# Sensory Neuron Expressed FcγRI Mediates Postinflammatory Arthritis Pain in Female Mice

**DOI:** 10.3389/fimmu.2022.889286

**Published:** 2022-06-27

**Authors:** Yan Liu, Michael J. Caterina, Lintao Qu

**Affiliations:** ^1^ Department of Neurosurgery, Neurosurgery Pain Research Institute, Johns Hopkins School of Medicine, Baltimore, MD, United States; ^2^ Solomon H. Snyder Department of Neuroscience, Johns Hopkins School of Medicine, Baltimore, MD, United States; ^3^ Department of Biological Chemistry, Johns Hopkins School of Medicine, Baltimore, MD, United States

**Keywords:** FcγRI, rheumatoid arthritis, pain, primary sensory neuron, collagen antibody-induced arthritis

## Abstract

Persistent arthritis pain after resolution of joint inflammation represents a huge health burden in patients with rheumatoid arthritis (RA). However, the underling mechanisms are poorly understood. We and other groups recently revealed that FcγRI, a key immune receptor, is functionally expressed in joint nociceptors. Thus, we investigated a potential role of sensory neuron expressed FcγRI in postinflammatory arthritis pain in a mouse model of collagen antibody-induced arthritis (CAIA). Here, we show that global deletion of *Fcgr1* significantly attenuated mechanical hyperalgesia in the ankle and hind paw of female mice in both inflammatory and postinflammatory phases of CAIA. No obvious differences in cartilage destruction were observed after resolution of joint inflammation between genotypes. *In situ* hybridization (ISH) revealed that a larger proportion of dorsal root ganglion (DRG) neurons expressed *Fcgr1* mRNA signal in the late phase of CAIA. Conditional deletion of *Fcgr1* in primary sensory neurons produced similar analgesic effects without affecting joint swelling. Knockdown of *Fcgr1* expression within DRG in the postinflammatory phase of CAIA alleviated persistent pain. Inflammation within DRG after resolution of joint inflammation in the CAIA model was evidenced by T cell and neutrophil infiltration and upregulated mRNA expression of numerous inflammatory mediators. Yet, such changes were not altered by genetic deletion of *Fcgr1*. We suggest that neuroinflammation within the DRG after resolution of joint inflammation might upregulate FcγRI signaling in DRG neurons. Sensory neuron expressed FcγRI thus merits exploration as a potential target for the treatment of arthritis pain that persists in RA patients in remission.

## Introduction

Rheumatoid arthritis (RA) is a common chronic autoimmune disease affecting 1% of the population across the world. Chronic pain is a dominant clinical symptom in RA patients, and poses an enormous health burden. Although joint inflammation has been considered as a critical contributor to RA pain, about 10-40% of RA patients continue to report joint pain even after joint inflammation is successfully controlled (1). In animal models of RA, hyperalgesia during the early inflammatory phase is attenuated by conventional therapeutics (nonsteroidal anti-inflammatory drugs, gabapentin, etanercept, the partial mu opioid receptor agonist buprenorphine), but hyperalgesia in the late postinflammatory phase is only modestly reduced by gabapentin, indicating that the pathways targeted by these drugs likely do not drive the chronification of arthritis pain (2, 3).

Despite significant advances in understanding RA pain mechanisms in the inflammatory phase, in fact, little effort has been undertaken to define the drivers of postinflammatory arthritis pain. A number of studies suggest that peripheral inflammation causes long-term alterations in nociceptive pathways at both peripheral and central levels (4, 5). In the spinal cord, astrocytes and microglia were activated in mice of both sexes after resolution of inflammation (4). Yet, inhibition of spinal glia activity reversed postinflammatory arthritis pain only in males but not in females (4). In addition, an inflammatory environment was observed within the DRG after resolution of joint inflammation, which drives continuous sensitization of nociceptors (6, 7). At a molecular level, voltage-gated Ca^2+^ channel (VGCC) subunit α2δ, P2X3, and certain cytokine receptors were upregulated in DRG neurons in the postinflammatory phase of collagen antibody-induced arthritis (CAIA) (5). In addition, RA is characterized by the production of autoantibodies and the formation of IgG immune complexes (IgG-IC). The level of certain autoantibodies remains elevated in subgroups of RA patients even after successful reversal of joint inflammation (8). FcγRI (also called CD64) is the sole high-affinity receptor that can bind both monomeric and polymeric IgG (9). FcγRI is most famously expressed in various immune cells (e.g., macrophages and T cells) and serves as a critical player in the regulation of immunity (10, 11). Yet, we and other groups recently discovered that FcγRI is also expressed in subsets of nociceptive DRG neurons (12, 13). Moreover, our recent study showed that neuronal FcγRI mediated arthritis pain *via* an inflammatory cell-independent mechanism during the inflammatory phase in mouse models of RA (14). Still, whether sensory neuron expressed FcγRI contributes to postinflammatory arthritis pain has remained entirely unexplored.

In the mouse model of CAIA, mechanical hyperalgesia persists even after resolution of joint inflammation (2). Thus, CAIA in mice faithfully recapitulates certain aspects of medically controlled RA or RA in remission in humans. Because of this feature, the CAIA model can be used to identify and evaluate candidate mechanisms driving postinflammatory arthritis pain. In this study, we test the hypothesis that sensory neuron expressed FcγRI mediates persistent joint pain after arthritis remission.

## Material and Methods

### Animals

Given the low incidence (~ 50%) of CAIA induction in BALB/c male mice, only BALB/c female mice that were 2 to 4 months old and 20 to 30 g body weight were used for this study (4). Animals were housed under a 14-hour light/10-hour dark cycle with ad libitum access to food and water. Breeders of global *Fcgr1* knockout (*Fcgr1^-/-^*) mice were provided by Sjef Verbeek (Leiden University Medical Center, Leiden, The Netherlands). *Fcgr1^-/-^*/BALB/c mice were obtained by backcrossing *Fcgr1^-/-^*/C57BL/6 mice at least five generations with BALB/c mice. *Fcgr1^fl/fl^* mice were previously generated by our laboratory using CRISPR/Cas9 genome editing (14). Specific deletion of *Fcgr1* expression in primary sensory neurons was achieved by mating *Fcgr1^fl/fl^* mice with a *PirtCre* mouse line (gift of Dr. Xinzhong Dong; Johns Hopkins University). This resulting mouse line was then backcrossed to the BALB/c genetic background at least five generations. All animal experimental procedures were approved by the Institutional Animal Care and Use Committee of Johns Hopkins University School of Medicine and were in accordance with the guidelines provided by the NIH and the International Association for the Study of Pain.

### Collagen Antibody-Induced Arthritis

CAIA was induced in female mice on a BALB/c background by intraperitoneal (i.p.) injection of an anti-CII arthritogenic cocktail (1.5 mg/mouse, 150 µl, Chondrex Inc, Woodinville, WA) on day 0 followed by i.p. injection of lipopolysaccharide (LPS, 25 µg/mouse, 100 µl in saline, Chondrex Inc, Woodinville, WA) on day 5 as described previously (2). Mice that receive saline i.p. on days 0 and 5 serve as controls. Arthritis score was used as an index of inflammation and disease activity, and evaluated by an investigator blinded to treatments and genotypes. Arthritis score was assigned based on a scoring protocol in which each swollen or red toe was given 1 point. A red or swollen base knuckle was given 1 point, a swollen ankle and/or wrist was given 5 points. The maximum score for each paw was 15 points, resulting in a maximum possible score of 60 points per mouse (2).

### Pain Behavioral Assessment

All behavioral measurements were performed on awake, age-matched littermates (2-4 months) by experimenters blinded to genotypes and treatments. Primary mechanical hyperalgesia in ankle joints was measured by applying ascending forces to the mouse ankle with calibrated electronic blunt forceps (Bioseb, Pinellas Park, FL) while mice were restrained with a cloth (14). The cutoff force was set at 350 g to avoid joint damage. The mechanical threshold was defined as the force at which the mouse withdrew its hindlimb forcefully or vocalized (14). The mechanical threshold in the joint was averaged over three measurements obtained at intervals of at least 5 min. Secondary mechanical hyperalgesia in the glabrous skin of hind paws was evaluated using von Frey monofilaments of different forces (0.04, 0.07, 0.16, 0.4, and 1.0 g). The frequency of paw withdrawal responses to ten applications of each filament was counted. Secondary thermal hyperalgesia in the glabrous hind paw skin was assessed by measuring withdrawal latency to noxious heat stimuli delivered using a radiant heat source (IITC Life Science Inc., Woodland Hills, CA). The cutoff latency was set at 15 s. Heat response latencies were averaged over three measurements obtained at intervals of at least 3 min.

### Quantitative Real-Time PCR

RNA was extracted from L3-L5 DRGs, synovium and lumbar spinal cord of mice using a RNeasy Lipid Tissue Mini Kit (Qiagen, Germantown, MD) and RNA quantity was measured using a NanoDrop 1000 (Thermo Fisher Scientific, Waltham, MA). RNA was then reverse-transcribed to complimentary DNA using the iScript cDNA synthesis kit (Bio-Rad, Hercules, CA) according to the manufacturer’s protocol. For relative quantification of mRNA, real time PCR was performed on a QuantStudio 3 real-time PCR system (Applied Biosciences Corp., Beverly Hills, CA) using the PowerUp SYBR Green Master Mix (Applied Biosciences Corp., Beverly Hills, CA). Each sample was run in duplicate. The expression levels of the target genes were quantified relative to the level of β-actin (Actb) gene expression using the 2^-ΔΔCT^ method. The sequence of primers used was as follows: *Fcgr1* forward: 5′-TGCTGGATTCTACTGGTGTGA-3′; *Fcgr1* reverse: 5′-AAACCAGACAGGAGCTGATGA-3′; *Il1b* forward: 5′-GGAGAACCAAGCAACGACAAAATA-3′; *Il1b* reverse: 5′-TGGGGAACTCTGCAGACTCAAAC-3′; *Il6* forward: 5′- AAAGAG TTGTGCAATGGCAATTCT-3′; *Il6* reverse: 5′-AAGTGCATCATCGTTGTTCATACA

-3′; *Mcp1* forward: 5′- GAAGCTGTAGTTTTTGTCACCA -3′; *Mcp1* reverse: 5′- TTCCTTCTTGGGGTCAGCAC-3′; *Cxcl1* forward: 5′- ATCCAGAGCTTGAAGGTGTTG -3′; *Cxcl1* reverse: 5′-GTCTGTCTTCTTTCTCCGTTACTT-3′; *Pirt* forward: TAGACGAGAGGTCTCCAGAGT; *Pirt* reverse: CCAGTTGCTTTTGGGTGTGG; *Actb* forward: 5′-CTGAATGGCCCAGGTCTGA-3′; *Actb* reverse: 5′-CCCTGGCTGCCTCAACAC-3′.

### siRNA Knockdown

ON-TARGETplus mouse CD64 siRNA SMARTPool (cat# L-040932-01) and non-targeting control pool (NC; cat# D-001810-10) were purchased from Dharmacon. siRNA was dissolved in 5% glucose and mixed with the *in vivo* transfection reagent (*In vivo*-jetPEI; N/P = 6; Polyplus Transfection Inc, New York, NY) and incubated at room temperature for 15 min according to the manufacturer’s instructions. A total of 2 µg of siRNA or NC in 10 µl volume was injected intrathecally (i.t.) into mice at L4/5 spinal levels once a day on days 49, 52 and 55 after the induction of CAIA. A valid spinal puncture and intrathecal delivery of the mixture was confirmed by a reflexive tail flick after needle entry into the subarachnoid space. Pain-related behaviors were assessed 72 hrs after each injection. At 72 hrs after the last injection, L3-5 DRGs and lumbar spinal cord was collected and subjected to qPCR to test the efficiency of *Fcgr1* knockdown.

### Immunohistochemistry and *In Situ* Hybridization

Anesthetized mice were transcardially perfused with phosphate-buffered saline, followed by 4% paraformaldehyde. L3-L5 DRGs were dissected, cryopreserved in 30% sucrose and cryosectioned at 12 µm. After blocking, sections were incubated with primary antibodies (**Table S1**) overnight at 4° C. Sections were then washed and incubated with complementary secondary antibodies (**Table S1**) at room temperature for 1 hr. ISH for *Fcgr1* mRNA was performed as described previously (14). SP6 transcribed antisense and T7 transcribed sense control probes were synthesized from mouse *Fcgr1* (NM_010186) cDNA clone (MR225268, OriGene) using one set of primers (forward, 5′-ATTTAGGTGACACTATAGAATCCTCAATGCCAAGTGACCC-3′; reverse, 5′-GCGTAATACGACTCACTATAGGGCGCCATCGCTTCTAACTTGC-3′). The specificity of these probes was validated in DRG sections of *Fcgr1^-/-^* mice in our previous study (14). The probes were then labeled using a digoxigenin (DIG) RNA labeling Kit (Roche Diagnostics Corp., Indianapolis, IN). After pre-hybridization, sections were hybridized with probes (2 ng/µl) at 60°C overnight and washed with 0.2x SSC at 62°C. To combine ISH with IHC, tissue sections undergoing ISH were incubated with sheep anti-DIG antibody (**Table S1**) and subjected to the standard IHC protocol as above. Images were captured using a confocal microscope (Nikon A1+; Nikon Metrology Inc, Brighton, MI). All images were analyzed using NIS elements in a blinded manner. At least three sections of L3-L5 DRGs per animal were analyzed for the quantification of DRG neuron subpopulations. To quantify *Fcgr1* expression, cell diameters were measured in a given DRG section using the polyline tool. Quantification of total neuron numbers was performed by counting NeuN positive neurons with clear nuclei. The proportion of *Fcgr1* positive neurons in each cell size population was calculated. For siRNA experiments, *Fcgr1* expression was quantified by measuring mean signal fluorescence intensity per neuron. For analysis of immune cell infiltration in DRG, 3-4 sections per animal of L3-L5 DRGs were imaged and the number or fluorescent intensity of cells immunopositive for each marker (CD3, Ly6C/G, and CD68) per unit area was counted. For each marker within a group, all parameters for image acquisition and analysis were kept constant across images.

### Histological Analysis

Total ankle joints were harvested from mice on day 56 after CAIA induction. Joints were decalcified in 0.5 mM ethylenediamine tetraacetic acid at 4°C for 3 weeks, then embedded in paraffin, and 5-μm coronal sections were cut. Sections were stained with Safranin-O and Fast Green for histological analysis. Destruction of the articular cartilage layers at the tibia and talus were scored on a scale from 0 (no erosion) to 3 (complete erosion of the calcified cartilage layer) (15). A total of 3 sections per joint from various depths were scored and results from all locations were averaged.

### Statistical Analysis

Data are presented as means ± SEM. A two-tailed Student’s t test was used to test the significance of differences between two groups. Comparisons for multiple groups or multiple time points were carried out using a two-way ANOVA for random measures or repeated measures followed by Bonferroni correction. P value less than 0.05 was considered significant. Sample sizes were chosen based on our pilot experiments, field conventions to accurately detect statistical significance, considering ethical animal use, experiment design, resource availability, and technical feasibility. Group size and statistical tests used for each comparison are noted in each figure legend. All statistical analyses were performed on GraphPad Prism 7.0 software.

## Results

### Global Deletion of Fcgr1 Attenuates Arthrits Pain and Joint Swelling in Female Mice With CAIA

**Figure 1:**
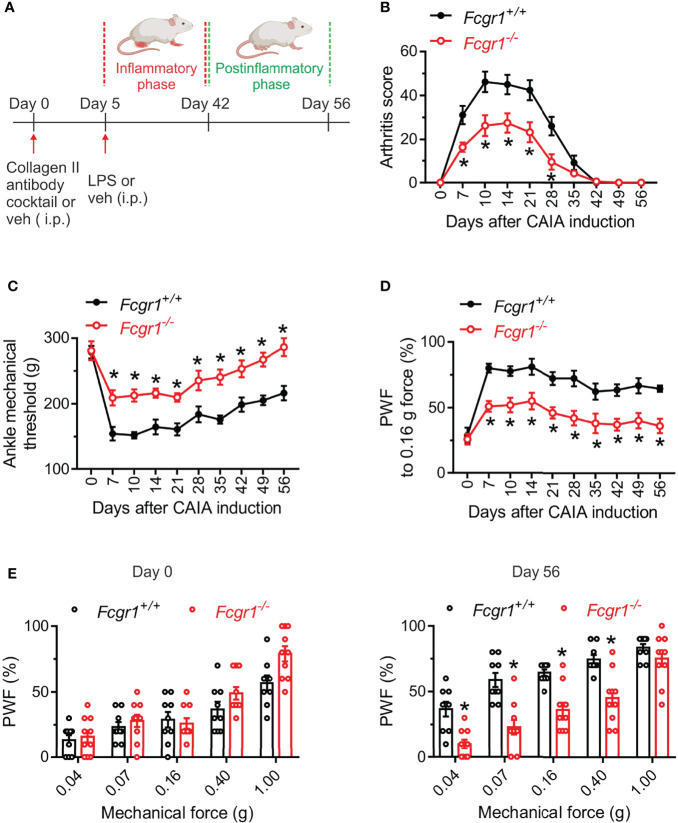
Global deletion of *Fcgr1* attenuates arthritis pain and joint swelling in female mice in the CAIA model. **(A)** Experimental diagram showing the induction of CAIA in female mice on a BALB/c background. **(B–D)** Time course of arthritis score **(B)**, mechanical threshold in the ankle **(C)**, and paw withdrawal frequency (PWF) in response to 0.16 g force in the hind paw **(D)**, in wildtype (*Fcgr1* ^+/+^; n = 9 mice) and global *Fcgr1* ^-/-^ (n = 10 mice) mice with CAIA. *p < 0.05, versus *Fcgr1* ^+/+^; two-way repeated measures ANOVA followed by Bonferroni correction. **(E)** Comparison of PWF in response to a series of mechanical forces (0.04, 0.07, 0.16, 0.40 and 1.0 g) in the hind paw of mice on days 0 and 56 after CAIA induction between *Fcgr1* ^+/+^ (n = 9) and *Fcgr1* ^-/-^ (n = 10) mice. *p < 0.05 versus *Fcgr1^+/+^*; two-way repeated measures ANOVA followed by Bonferroni correction.

Although our recent study showed that FcγRI mediated arthritis pain in several inflammatory arthritis models (14, 16), much less is known about its potential roles in arthritis pain and disease activity in the CAIA model. Considering that the CAIA model allows us to define postinflammatory mechanisms of arthritis pain, we compared pain related behaviors and arthritis scores between wildtype (*Fcgr1* ^+/+^) and *Fcgr1* ^-/-^ mice in both inflammatory and postinflammatory phases of CAIA. Consistent with previous studies (2, 5), *Fcgr1* ^+/+^ mice subjected to CAIA developed obvious joint swelling and redness (indicated by arthritis score) on day 7, peaking around day 14 and gradually resolving by around day 42 (**Figures 1A, B**). In contrast, mechanical hyperalgesia in both the ankle (**Figure 1C**) and hind paw (**Figures 1D, E**) of mice maintained evident during and beyond the resolution of overt joint swelling in the CAIA model (from day 6 to day 56; **Figure 1**). We refer to the period of hyperalgesia with visible signs of joint swelling as the ‘inflammatory phase’ (days 7-35) and that without obvious joint swelling as the ‘postinflammatory phase’ (days 42-56). Arthritis scores in *Fcgr1^-/-^* mice were markedly attenuated in the inflammatory phase compared to wildtype controls (**Figure 1B**). Strikingly, despite apparently normal ankle and hind paw mechanical sensitivity prior to the induction of arthritis, global *Fcgr1^-/-^* mice also exhibited less mechanical hyperalgesia in the ankle and hind paw, compared with *Fcgr1* ^+/+^ littermates, throughout both the inflammatory and postinflammatory phases of CAIA (**Figures 1C–E**).

**Figure 2:**
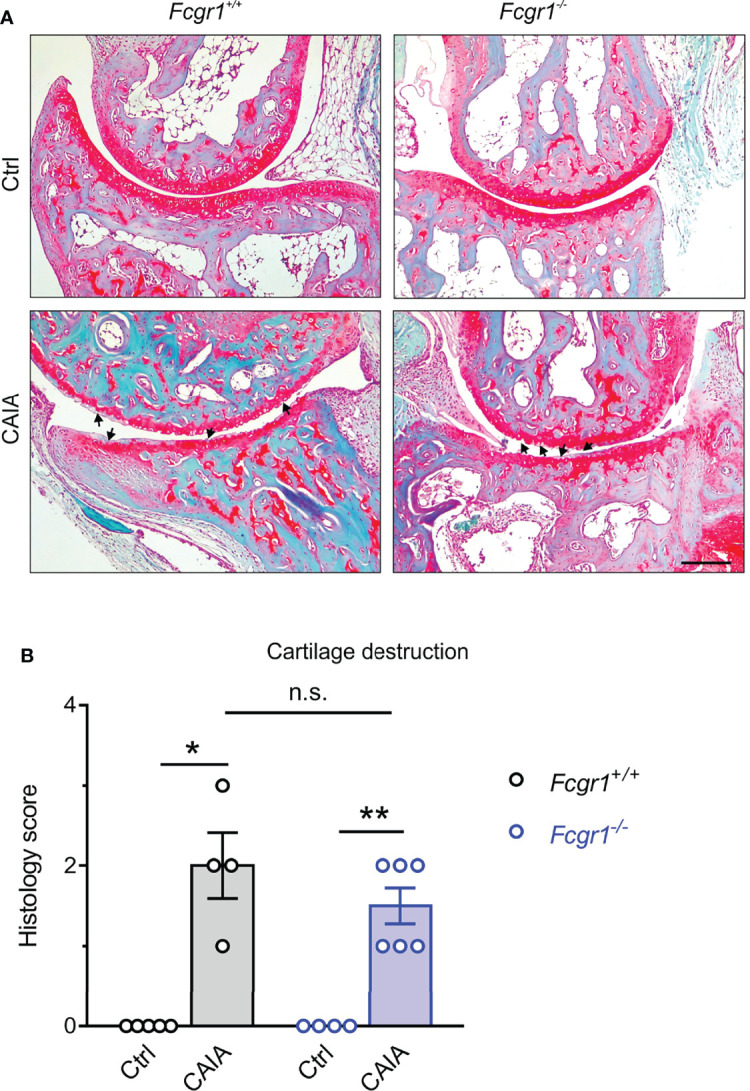
FcγRI is not necessary for cartilage destruction in the postinflammatory phase of CAIA. **(A)** Representative images of Safranin O/fast green-stained ankle joints from *Fcgr1^+/+^* and *Fcgr1^-/-^* mice on day 56 after CAIA. Arrowheads indicated cartilage destruction. Scale bar, 200 μm. **(B)** No obvious differences in the histology score for cartilage destruction were observed at day 56 after CAIA between genotypes. n = 4 - 6 mice/group; *p < 0.05; **p < 0.01 versus control (Ctrl), two-way ANOVA followed by Bonferroni correction. n.s., not significant.

FcγRI has been implicated in cartilage and bone damage in antigen-induced arthritis (AIA) and collagen II-induced arthritis (CIA) models (12, 13). To determine whether the apparent antihyperalgesic effects of *Fcgr1* knockout were attributable to a possible reduction in CAIA-induced cartilage destruction, we performed Safranin-O/fast green staining on mouse ankle joint sections (**Figure 2**). Safranin-O/fast green staining analysis revealed obvious cartilage destruction in both genotypes on day 56 after CAIA induction (**Figure 2A**). However, the extent of cartilage damage was not significantly different between genotypes in the CAIA model (**Figure 2B**).

### Conditional Knockout of Fcgr1 in Primary Sensory Neurons Alleviates Arthritis Pain in the CAIA Model

**Figure 3:**
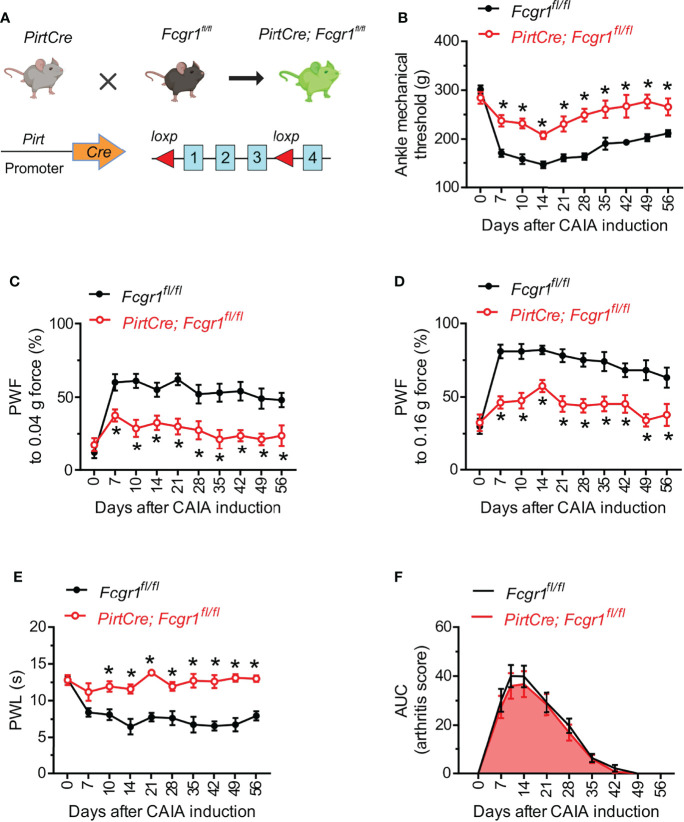
Neuronal FcγRI mediates arthritis pain in the late phase of the CAIA model. **(A)** Strategy for the generation of primary sensory neuron specific *Fcgr1* knockout mice. Two *loxp* sites were inserted 5’ to exon 1 and 3’ to exon 3 of the *Fcgr1* gene, respectively. Deletion of the *Fcgr1* gene in primary sensory neurons was achieved by mating *Fcgr1^fl/fl^* mice with a *PirtCre* mouse line. **(B, E)** Time course of ankle mechanical threshold **(B)**, paw withdrawal frequency (PWF) to 0.04 g **(C)** and 0.16 g force **(D)**, and paw withdrawal latency (PWL) to radiant heat in the hind paw **(E)** in *PirtCre- Fcgr1^fl/fl^* mice and *Fcgr1^fl/fl^* control littermates following CAIA. n = 8-10 mice per group for **(B–D)**; n = 5-6 mice per group for **(E)**; *p < 0.05 versus *Fcgr1^fl/fl^* controls; two-way repeated measures ANOVA followed by Bonferroni correction. **(F)** Time course of arthritis score (area under the curve; AUC) of *PirtCre-Fcgr1^fl/fl^* mice and *Fcgr1^fl/fl^* control littermates in the CAIA model. n = 14-15 mice per group; P > 0.05 versus *Fcgr1^fl/fl^*; two-way repeated measures ANOVA followed by Bonferroni correction.

Given that neuronal FcγRI can regulate the excitability of primary sensory neurons (13, 14), we next investigated whether neuronally expressed FcγRI contributes to postinflammatory arthritis pain in the CAIA model. *Fcgr1^fl/fl^* mice were crossed with the *PirtCre* line to selectively omit FcγRI expression from primary sensory neurons (**Figure 3A**). The specific loss of FcγRI expression in primary sensory neurons was validated in our recent studies (14). In both inflammatory and postinflammatory phases of CAIA, *PirtCre-Fcgr1^fl/fl^* mice displayed less mechanical hyperalgesia in the ankle and hind paw, and less heat hyperalgesia in the hind paw, compared to *PirtCre* negative controls (**Figures 3B–E**). However, unlike our findings in global *Fcgr1^-/-^* mice, sensory neuron-specific *Fcgr1* deletion resulted in no significant differences in arthritis scores, compared to *PirtCre* negative controls, in the inflammatory phase of CAIA (**Figure 3F**). Interestingly, CAIA caused a significant downregulation of *Pirt* mRNA expression in the DRG on day 56 after challenge (**Supplementary Figure 1**). However, disruption of the *Fcgr1^fl/fl^* alleles would have occurred earlier in life, and therefore should not be affected by this phenomenon.

### Knockdown of Fcgr1 Expression in the DRG in the Postinflammatory Phase of CAIA Attenuates Persistent Pain

**Figure 4:**
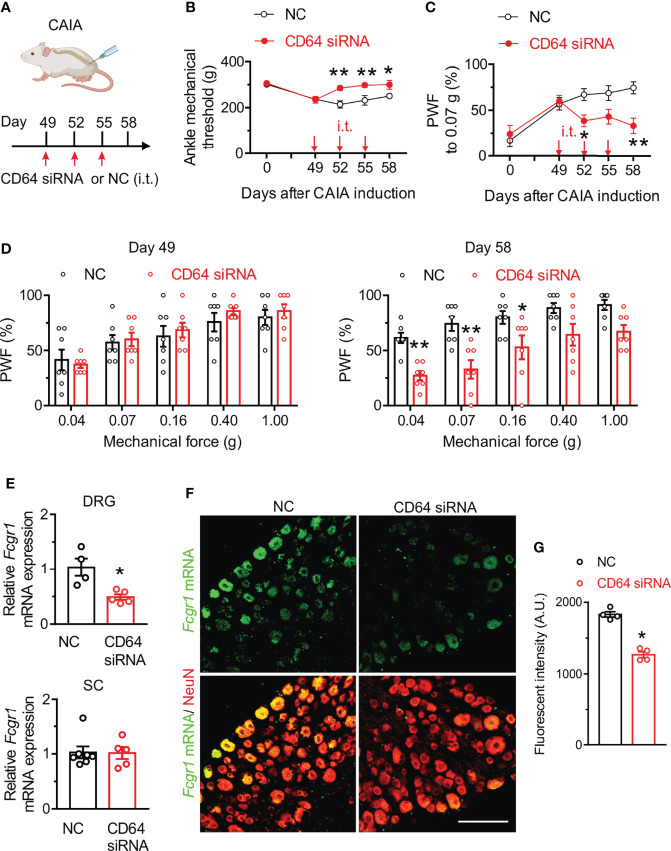
CD64 siRNA knockdown in the DRG after resolution of joint inflammation alleviates persistent pain in the CAIA model. **(A)** Experimental schematic showing intrathecal (i.t.) administration protocol of CD64 siRNA (2 µg; 10 µl) or a negative control (NC; 2 µg; 10 µl) in mice subjected to CAIA. Pain-like behaviors were evaluated 3 days after each injection. **(B, C)** Time course of mechanical threshold in the ankle **(B)**, paw withdrawal frequency (PWF) in response to 0.07 g force **(C)** in the hind paw in NC- and CD64 siRNA- treated mice with CAIA. n = 7 mice per group; *p < 0.05, **p < 0.01 versus NC; two-way repeated measures ANOVA followed by Bonferroni correction. **(D)** Comparison of PWF in response to a series of mechanical forces (0.04, 0.07, 0.16, 0.40 and 1.0 g) in the hind paw of mice on days 49 and 58 after CAIA induction between NC- and CD64 siRNA-treated mice. n = 7 mice per group. *p < 0.05, **p< 0.01 versus NC, two-way repeated measures ANOVA followed by Bonferroni correction. **(E)** qPCR analysis of *Fcgr1* mRNA expression in L3-L5 DRGs (n = 4-5 mice per group) and spinal cord (SC; n = 5-7 mice per group) in NC and CD64 siRAN treated mice on day 58 after CAIA induction. *p < 0.05 versus NC; unpaired Student’s t test. **(F)** Representative L4 DRG ISH image for *Fcgr1* mRNA signal and immunostaining for NeuN. Scale bar: 100 µm. **(G)** Quantification of *Fcgr1* mRNA expression in DRG neurons of either NC or CD64 siRNA-treated mice. n = 4 mice per group; *p < 0.05 versus NC; unpaired Student’s t test.

To further determine the contribution of FcγRI to postinflammatory arthritis pain, we evaluated whether specific silencing of *Fcgr1* in the DRG after resolution of joint inflammation was sufficient to alleviate persistent pain in the CAIA model. To test this possibility, we delivered CD64 siRNA or a non-targeting siRNA negative control by i.t. injections into wildtype mice on days 49, 52 and 55 after CAIA induction, a time period after joint inflammation was resolved. Mechanical hyperalgesia was assessed 72 hrs after each injection (**Figure 4A**). CD64 siRNA significantly increased mechanical threshold in the ankle of mice on all testing days, compared to the negative control (**Figure 4B**). Similarly, CAIA mice treated with CD64 siRNA exhibited less secondary mechanical hyperalgesia in the hind paws than those treated with the negative control (**Figure 4C, D**). In addition, we harvested L3-L5 DRGs after behavioral testing on day 58 of CAIA induction and found a significant decrease of *Fcgr1* mRNA expression in the DRG but not in spinal cord after CD64 siRNA injection compared to negative control injection (**Figure 4E**). ISH assay confirmed a significant reduction in *Fcgr1* mRNA signal in DRG neurons in CD64 siRNA-treated mice compared to that in negative control-treated animals (**Figures 4F, G**).

### Fcgr1 mRNA Expression in Mouse DRG Is Upregulated in the Late Phase of CAIA

**Figure 5:**
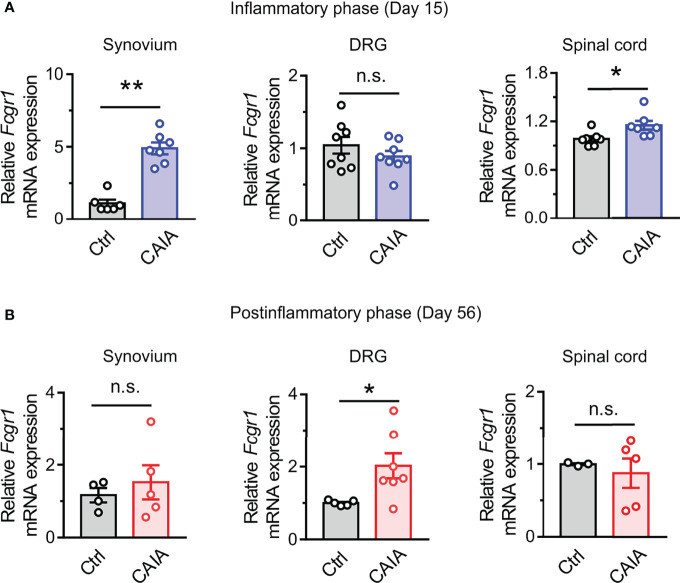
*Fcgr1* mRNA expression changes dynamically over the course of CAIA. **(A, B)** qPCR analysis of *Fcgr1* mRNA expression normalized to that of *Actb* in the DRG, synovium, and spinal cord of control (Ctrl) and CAIA mice on days 15 (**A**; n = 6-8 mice per group) and (**B**; n = 3-7 mice per group) 56 after immunization. *p < 0.05, **p < 0.01 versus Ctrl unpaired Student’s t test. n.s., not significant.

**Figure 6:**
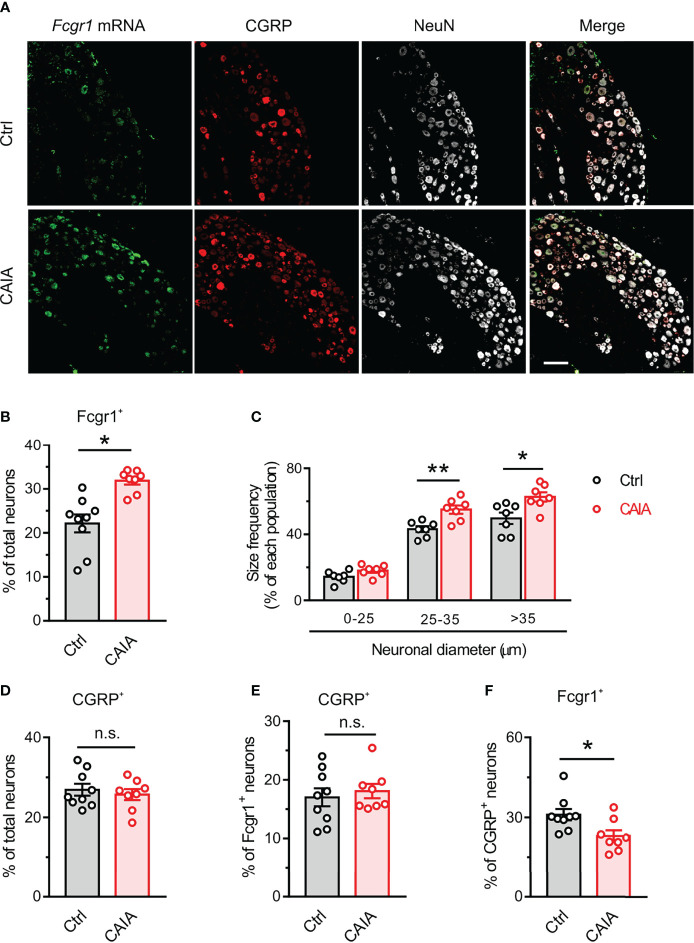
FcγRI mRNA expression is upregulated in DRG neurons in the postinlfammatory phase of CAIA. **(A)** Representative lumbar DRG ISH for *Fcgr1* and immunostaining for CGRP and NeuN from control and CAIA mice on day 56 after challenge. Scale bar, 50 μm. **(B)** Percentage of *Fcgr1* ^+^ neurons among all DRG neurons in control (Ctrl) and CAIA mice. n = 7-8 mice per group; *p < 0.05 versus control; unpaired Student’s t test. **(C)** Size frequency distribution of *Fcgr1* mRNA expression in each cell size population in DRG neurons of control and CAIA mice on day 56 after immunization. n = 7 mice per group; *p < 0.05, **p < 0.01 versus Ctrl; unpaired Student’s t test. **(D)** Percentage of CGRP^+^ neurons among all DRG neurons in control and CAIA mice. n = 8-9 mice per group; p > 0.05 versus Ctrl; unpaired Student’s t test. **(E)** Percentage of *Fcgr1^+^* neurons among CGRP^+^ neurons in control and CAIA mice. n = 8-9 mice per group; P > 0.05 versus Ctrl; unpaired Student’s t test. **(F)** Percentage of CGRP^+^ neurons among *Fcgr1^+^* neurons in control and CAIA mice. n = 8-9 mice per group; *p < 0.05 versus Ctrl; unpaired Student’s t test. n.s., not significant.

Our previous studies revealed that *Fcgr1* mRNA expression was upregulated in the DRG during inflammation in both AIA and CIA models (14, 16). However, whether such changes occur in the CAIA model remained unknown. Therefore, we performed qPCR on the DRG from wildtype mice to assay for the alterations of *Fcgr1* mRNA expression on days 15 (inflammatory phase) and 56 (postinflammatory phase) after CAIA induction. On day 15 after immunization, the *Fcgr1* mRNA expression level was significantly upregulated in the synovium and spinal cord but not in the DRG of mice with CAIA compared to vehicle treated animals (**Figure 5A**). By contrast, on day 56 after CAIA induction, upregulated levels of *Fcgr1* mRNA expression were observed in the DRG but not the synovium and spinal cord in the CAIA group compared to control mice (**Figure 5B**). To further define the changes in *Fcgr1* mRNA expression profile in the DRG at late stages of CAIA, and to determine whether these changes occurred within sensory neurons, we carried out ISH and IHC assays on the DRG on day 56 after CAIA induction. We revealed that a larger proportion of DRG neurons expressed *Fcgr1* mRNA in CAIA mice compared control animals (**Figures 6A, B**). Furthermore, these alterations were statistically significant in medium and large diameter DRG neurons but not in small diameter neurons. (**Figure 6C**). The specificity of *Fcgr1* ISH probes was validated in our recent studies (data not shown) (14). In agreement with previous studies (5), the number of CGRP^+^ neurons were not altered in the DRG at the late phase of CAIA (**Figure 6D**). Among *Fcgr1* mRNA expressing neurons, we also observed no significant alterations in the percentage of CGRP^+^ neurons during the postinflammatory phase of CAIA (**Figure 6E**). Surprisingly, however, the proportion of CGRP^+^ DRG neurons that also expressed *Fcgr1* mRNA was significantly reduced on day 56 after CAIA induction (**Figure 6F**), suggesting that an increase in *Fcgr1* mRNA expression may exclusively occur in non-CGRP expressing neurons.

### FcγRI Does Not Contribute to Immune Cell Infiltration Within the DRG in the Postinflammatory Phase of CAIA

**Figure 7:**
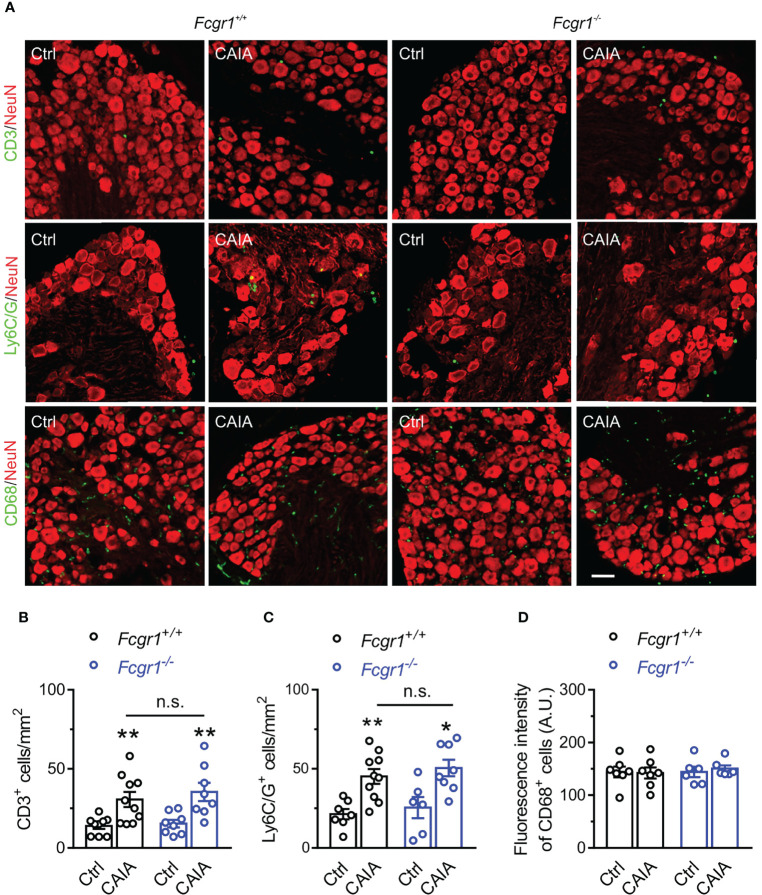
FcγRI does not contribute to immune cell infiltration in mouse DRG in the late phase of CAIA. **(A)** Representative image of mouse DRG sections from *Fcgr1^+/+^* and *Fcgr1^-/-^* mice on day 56 after CAIA. DRG sections were stained for CD3, Ly6C/G, CD68 and NeuN. Scale bar, 50 μm. **(B–D)** Quantification showed an increase in the number of CD3^+^ (**B**; n = 8-10 mice/group) and Ly6C/G^+^ (**C**; n = 6-10 mice/group) cells per unit area but not the mean fluorescent intensity of CD68^+^ (**D**; n = 6-7 mice/group) per unit area in the DRG of either genotype on day 56 after CAIA induction. Yet, no obvious differences in CD3^+^ or Ly6C/G^+^ cell infiltration were observed between genotypes. *p < 0.05, **p < 0.01 versus Ctrl, two-way ANOVA followed by Bonferroni *post hoc* test. n.s., not significant.

**Figure 8:**
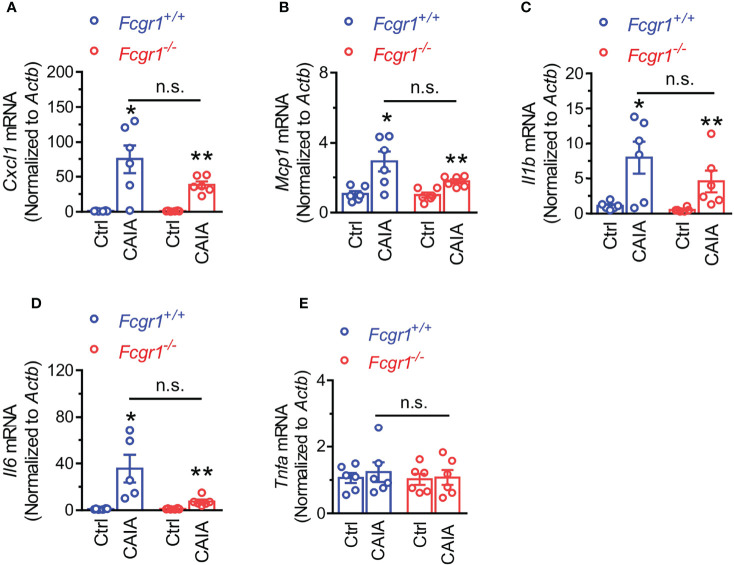
Genetic deletion of *Fcgr1* does not affect the upregulation of mRNA expression of proinflammatory mediators in the DRG after resolution of inflammation in the CAIA model. **(A–E)** qPCR analysis of the mRNA expression (normalized to that of *Actb*) of *Cxcl1* **(A)**, *Mcp1* **(B)**, *Il1b* **(C)**, *Il6* **(D)**, and *Tnfa* **(E)** in the DRG of *Fcgr1^+/+^* and *Fcgr1^-/-^* mice on day 56 after immunization. n = 5-6 mice/group; *p < 0.05, **p < 0.01 versus control (Ctrl); two-way ANOVA followed by Bonferroni *post hoc* test. n.s., not significant.

Considering that immune cell infiltration within the DRG has been implicated in postinflammatory arthritis pain (6, 7), we next asked whether FcγRI is necessary for immune cell infiltration within the DRG in the postinflammatory phase of CAIA. On day 56 after CAIA induction, IHC analysis showed that markers for T cells (CD3; **Figures 7A, B**), and neutrophils and monocytes (Ly6C/G; **Figures 7A, C**) were increased in the DRG whereas the marker for macrophages (CD68; **Figures 7A, D**) was not changed. However, none of the CAIA-induced increases in any of these markers were significantly different between genotypes (**Figures 7B, C**). To further determine whether there is an inflammatory environment in the DRG in the late phase of the CAIA model, we measured mRNA expression of a number of cytokines and chemokines in wildtype mice on day 56 after CAIA induction. Among all genes tested, the expression levels of *Cxcl1*, *Mcp1*, *Il-1b*, and *Il-6* were significantly upregulated in the DRG after arthritis remission (**Figures 8A–D**) whereas no significant changes in *Tnfα* mRNA expression in the DRG were observed (**Figure 8E**). However, while there were trends towards lower levels of upregulation of *Cxcl1*, *Mcp1*, *Il-1b*, and *Il-6* in global *Fcgr1^-/-^* mice, no significant differences in the extent of the upregulation of these inflammatory mediators were observed between genotypes (**Figures 8A–D**).

## Discussion

Arthritis pain often persists in a significant subpopulation of RA patients even after joint inflammation has resolved. Although central mechanisms of postinflammatoy arthritis pain are well defined (4, 5), the studies on peripheral mechanisms are quite sparse. In the present study, we have focused on the late phase of the CAIA model when joint inflammation has resolved but arthritis pain persists. Consistent with prior findings in mouse AIA and K/BxN serum transfer models (6, 7), we have identified an inflammatory state within DRG after arthritis remission. We also provide new evidence for the involvement of sensory neuron expressed FcγRI in mechanical hypersensitivity in both inflammatory and postinflammatory phases of CAIA.

FcγRI is widely expressed in immune cells (e.g., macrophages and T cells) and plays a key role in the pathogenesis of RA (17). In RA patients, FcγRI is expressed *de novo* in the synovium (18) and its expression level in neutrophils, monocytes and synovial macrophages is upregulated (19–21). The pathogenicity of FcγRI has been confirmed in animal models of AIA and CIA (21–23). Our previous work has demonstrated the contribution of FcγRI to arthritis pain in several murine models of inflammatory arthritis (14, 16). We and other groups have reported that FcγRI is also present in primary sensory neurons besides immune cells (13, 24, 25). Moreover, we showed that neuronally expressed FcγRI mediated the sensitization of joint nociceptors and arthritis pain during inflammation in animal models of RA through a mechanism independent of inflammation (14). The present study extended previous studies by showing that FcγRI signaling, particularly in primary sensory neurons, contributes to persistent arthritis pain observed after resolution of joint inflammation. First, global deletion of *Fcgr1* alleviated mechanical hyperalgesia in the ankle and hind paws of mice in both inflammatory and postinflammatory phases of the CAIA model. Since cold hyperalgesia occurred at the same time as joint inflammation but did not persist beyond the signs of arthritis (2, 3), we did not evaluate those behaviors in this study. Second, the contribution of neuronal FcγRI to postinflammatory pain was validated by use of conditional *Fcgr1* knockout mice, in which *Fcgr1* is specifically deleted in primary sensory neurons. Indeed, conditional deletion of *Fcgr1* produced similar antihyperalgesic effects as global *Fcgr1* knockout. However, unlike our findings in global *Fcgr1^-/-^* mice, our conditional knockout data suggest that neuronal FcγRI might not be involved in joint swelling in the CAIA model, at least as reflected by arthritis score. Further work is warranted to define any contributions of neuronal FcγRI to less overt joint inflammatory processes in the setting of RA, using more highly sensitive histological and biochemical assays, including flow cytometry and hematoxylin and eosin stain (H&E) staining. Lastly, qPCR revealed that the upregulated *Fcgr1* mRNA expression differed across anatomical loci over the course of CAIA. During the inflammatory phase of CAIA, we observed an upregulation of *Fcgr1* mRNA expression in the synovium and spinal cord but not in the DRG. By contrast, upregulation of *Fcgr1* mRNA expression was observed only in the DRG but not in the synovium and spinal cord after arthritis remission in the CAIA model. ISH analysis further supported the upregulation of *Fcgr1* mRNA expression in DRG neurons in the postinflammatory phase of CAIA. Moreover, these changes mainly occurred in medium and large diameter DRG neurons. Unlike during inflammatory stages of AIA, where there is an increase in the proportion of FcγRI expressing neurons that also express CGRP (14), in the late phase of the CAIA model, we did not observe such increases. In fact, the overall number of CGRP^+^ cells also expressing FcγRI was reduced at this time point, suggesting that an increase in *Fcgr1* mRNA expression may occur exclusively in non-CGRP expressing neurons. Considering that neuronal FcγRI is important to the regulation of the excitability of primary sensory neurons (14), it is possible that the observed upregulation of *Fcgr1* mRNA expression in DRG neurons may contribute to continuous peripheral sensitization and postinflammatory arthritis pain. This notion was further supported by use of selective CD64 siRNA. Intrathecal delivery of CD64 siRNA into CAIA mice in the postinflammatory phase downregulated *Fcgr1* mRNA expression in the DRG neurons and alleviated persistent arthritis pain. This is consistent with the possibility that the antihyperalgesic effect of CD64 siRNA is mediated, at least in part, by sensory neuron expressed FcγRI. Since FcγRI is also expressed in immune cells (17), however, we cannot rule out non-neuronal effects of CD64 siRNA in this experiment. In addition, considering that CD64 siRNA was administered through intrathecal delivery,we also cannot completely exclude a central action of CD64 siRNA.

Cartilage and bone damage appears to be a key driver of arthritis pain in the absence of visible joint swelling (i.e., osteoarthritis pain) (26). Clinical studies suggest that the destruction of cartilage and bone persists in RA patients after resolution of joint inflammation (27, 28). FcγRI has been implicated in cartilage destruction and bone erosion during the inflammatory phase in animal models of CIA and AIA (22, 23). Given that FcγRI is a key immune receptor expressed in a variety of immune cells (29), FcγRI might indirectly regulate bone erosion *via* the recruitment and activation of immune cells during RA (30). In addition, FcγRI is also expressed in preosteoclasts and osteoclasts (31, 32). Direct activation of FcγRI in these cells increases both osteoclast differentiation and function (31, 32). Consistent with clinical studies, the present study showed obvious cartilage destruction in the postinflammatory phase of the CAIA model. However, FcγRI is not necessary for these anatomical changes since genetic deletion of *Fcgr1* did not alter the degree of cartilage damage following arthritis remission.

Neuroinflammation within the DRG has been implicated in the development and maintenance of arthritis pain during the inflammatory phase (33, 34). Recent studies revealed persistent immune cell infiltration in the DRG after joint inflammation was resolved in AIA and K/BxN serum transfer models (6, 7). Moreover, increased numbers of activated resident macrophages in the DRG released TNF, which may drive continuous sensitization of primary sensory neurons and the maintenance of persistent joint pain following the remission of AIA (7). However, in the CAIA model, we did not observe obvious macrophage infiltration within the DRG after joint inflammation was resolved. By contrast, our results revealed infiltration of T cells and neutrophil and/or monocytes and elevated expression level of a variety of proinflammatory chemokines and cytokines in the DRG after arthritis remission. Although FcγRI is critical to the regulation of immune responses (11), the present study found that genetic deletion of *Fcgr1* had no significant effects on immune cell infiltration or the extent of the upregulation of inflammatory mediators in the DRG after resolution of joint inflammation. Thus, it is unlikely that FcγRI signaling acts as a major upstream mechanism for immune cell infiltration and release of proinflammatory mediators in the DRG after arthritis remission. By contrast, FcγRI may function as a downstream target of proinflammatory mediators. These inflammatory mediators within the DRG might upregulate FcγRI expression in primary sensory neurons and/or enhance the affinity of neuronal FcγRI for IgG-IC, leading to peripheral sensitization and persistent arthritis pain after arthritis remission (35, 36).

The endogenous activators of FcγRI during postinflammatory arthritis remain unclear. Conventional wisdom has held that autoantibodies revert to negative titers after RA patients achieve disease-modifying antirheumatic drug -free remission (37, 38). However, a recent clinical study revealed no obvious association between disease remission and reversion to autoantibody negativity (8). Anti-citrullinated protein antibodies (ACPA) IgG levels were not significantly altered in a subpopulation of RA patients in remission (8). ACPA IgG together with their citrullinated antigens might form IgG-IC to activate joint nociceptors to trigger arthritis pain *via* their interaction with neuronally expressed FcγRI (39). Considering that FcγRI plays an important role in the regulation of substance P and CGRP release from DRG neurons induced by IgG-IC (12, 24), neuronal FcγRI may contribute to postinflammatory arthritis pain through peripheral and central mechanisms by modulating the release of these pain mediators. Further investigations are needed to test this possibility.

This study has some limitations. We did not evaluate pain-related behaviors in male mice due to the low incidence of CAIA induction in males [4]. Considering that sexual dimorphism was observed in central mechanisms of postinflammatory arthritis pain [4], further investigations are warranted to assay for potential sex differences in the role of peripheral FcγRI in persistent joint pain after arthritis remission. Since Cre rather than inducible CreER mice were used in this study, the observed diminished mechanical pain hypersensitivity in *Fcgr1* knockout mice after arthritis remission may be secondary to antihyperalgesic effects of FcγRI in the inflammatory phase of arthritis. Further investigation is required to determine whether inducible deletion of the *Fcgr1* gene in primary sensory neurons after arthritis remission attenuates persistent joint pain.

In conclusion, our results provide novel evidence that FcγRI expressed in primary sensory neurons contributes to the maintenance of postinflammatory arthritis pain. Although FcγRI is not necessary for the development of the late state of neuroinflammation in the DRG, the prolonged neuroinflammation within the DRG after arthritis remission might upregulate FcγRI signaling in primary sensory neurons, leading to peripheral sensitization and persistent arthritis pain. We suggest that neuronal FcγRI may represent a new candidate therapeutic target for the treatment of persistent arthritis pain in RA patients with low disease activity and/or in remission.

## Data Availability Statement

The raw data supporting the conclusions of this article will be made available by the authors, without undue reservation.

## Ethics Statement

The animal study was reviewed and approved by the Institutional Animal Care and Use Committee of Johns Hopkins University School of Medicine.

## Author Contributions

YL performed IHC, joint histology staining, quantitative RT-PCR, and *in situ* hybridization experiments, and analyzed the data. MC facilitated experimental design and analysis, and revised the manuscript. LQ conceived the project, designed the experiments, conducted behavioral tests, analyzed the data, and wrote the manuscript. All authors contributed to the article and approved the submitted version.

## Funding

This work is supported by the National Institutes of Health (AR072230; to LQ), Johns Hopkins Blaustein Pain Research Grant (to LQ), Johns Hopkins Catalyst Award (to LQ) and the Neurosurgery Pain Research Institute at Johns Hopkins University.

## Conflict of Interest

The authors declare that the research was conducted in the absence of any commercial or financial relationships that could be construed as a potential conflict of interest.

## Publisher’s Note

All claims expressed in this article are solely those of the authors and do not necessarily represent those of their affiliated organizations, or those of the publisher, the editors and the reviewers. Any product that may be evaluated in this article, or claim that may be made by its manufacturer, is not guaranteed or endorsed by the publisher.

## Supplementary Material

Supplementary Figure 1 Pirt mRNA expression is downregulated in the DRG in the posinflammatory phase of CAIA. qPCR analysis of Pirt mRNA expression in L3-L5 DRGs of control (Ctrl) and CAIA mice on days 56 after immunization. n = 8 mice per group. p < 0.01 versus Ctrl, unpaired Student’s t-test.
