# Supplementary figures and images for: Sensory Neuron Expressed FcγRI Mediates Postinflammatory Arthritis Pain in Female Mice
Source: Front Immunol. 2022 Jun 27;13:889286. doi: 10.3389/fimmu.2022.889286 (PMC9271677; doi:10.3389/fimmu.2022.889286)

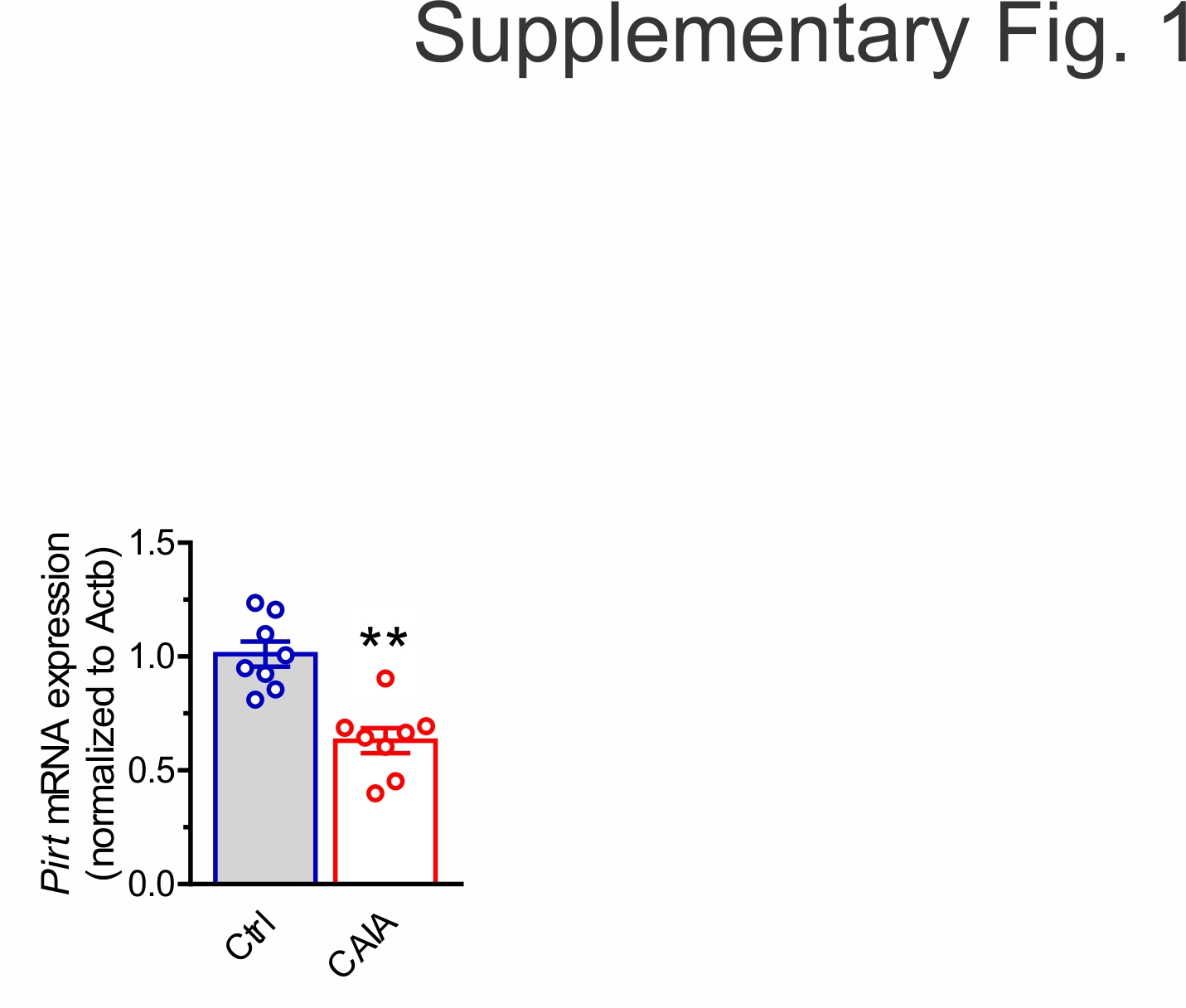

Supplement: Supplementary Figure 1 — Pirt mRNA expression is downregulated in the DRG in the posinflammatory phase of CAIA. qPCR analysis of Pirt mRNA expression in L3-L5 DRGs of control (Ctrl) and CAIA mice on days 56 after immunization. n = 8 mice per group. p < 0.01 versus Ctrl, unpaired Student’s t-test. [file Image_1.jpeg]
