# Supplementary material for: Sensory Neuron Expressed FcγRI Mediates Postinflammatory Arthritis Pain in Female Mice
Source: Front Immunol. 2022 Jun 27;13:889286. doi: 10.3389/fimmu.2022.889286 (PMC9271677; doi:10.3389/fimmu.2022.889286)
Supplement: Supplementary file 2 [file Table_1.docx]

**Table S1. List of antibodies**

| Name | Dilution | | Source | | Identifier | |
| --- | --- | --- | --- | --- | --- | --- |
| Rat anti-CD3  Rat anti-CD68  Rat anti-Ly6C/G  Chicken anti-NeuN  Rabbit anti-CGRP  Sheep anti-DIG  Goat anti-rat IgG Alexa 488  Donkey anti-rabbit IgG Cy3  Donkey anti-rabbit IgG Alexa 647  Donkey anti-sheep IgG Alexa 488  Donkey anti-chicken IgG Cy3 | | 1:50  1:500  1:500  1:200  1:500  1:200  1:500  1:500  1:500  1:500  1:500 | | BD Biosciences  BioLegend  BD Pharmingen  Aves  Immunostar  Roche  Invitrogen  Jackson ImmunoResearch labs  Jackson ImmunoResearch labs  Abcam Jackson ImmunoResearch labs | | Cat# 555273  Cat#137001  Cat# 550291  Cat# NUN  Cat# 24112  Cat#1133089001  Cat# A11006  Cat# 711165152  Cat# 711-605-152  Cat# Ab15077  Cat# 703165155 |
